# Mesothelin as a Signal Pathways and Epigenetic Target in Cancer Therapy

**DOI:** 10.3390/cancers17071118

**Published:** 2025-03-26

**Authors:** Seema Kumari

**Affiliations:** Department of Biotechnology, Dr. B.R Ambedkar University, Srikakulam 532410, Andhra Pradesh, India; seemakumarisingh@gmail.com

**Keywords:** mesothelin, CA125/MUC16, cancer progression, epigenetic regulation, MAPK pathways

## Abstract

Mesothelin (MSLN), a glycoprotein-based tumor antigen, is elevated in several malignancies and it is related to a poor prognosis, as it enhances tumor aggression, dissemination and chemotherapy resistance. MSLN plays a crucial role in epigenetic and signal pathway regulation and it can be an important biomarker. MSLN targeting is in particular, associated with CA125/MUC16, which offers the potential to improve lung, pancreatic, colon and ovarian cancer detection as well as therapeutic strategies. MSLN-targeted therapies have shown favorable results, such as CAR NK cells, 227Th conjugate and CAR-T cells, which target mesothelin. Significant advancements can be achieved with novel techniques, such as mesothelin-targeting BiTEs and simultaneous CAR-T cells. Immunotherapies targeting mesothelin have the potential to completely transform the way cancer is therapy in patients with limited options. To fully comprehend the mechanisms of MSLN, more investigation is required to explore its role in cancer for improved patient outcomes. The complex control, cellular functions and clinical significance of MSLN in the advancement of cancer are highlighted in this review.

## Simple Summary

Mesothelin, a glycoprotein-based tumor antigen is elevated in several malignancies. It contributes to tumor aggression, metastasis, chemotherapy resistance and is linked with poor prognosis. MSLN expression is regulated by epigenetic changes, which control various proliferation signal pathways. MSLN can be used as biomarker for diagnosis and MSLN-targeted therapy has provided favorable results in various carcinogenesis.

## 1. Introduction to Mesothelin

**Figure 1:**
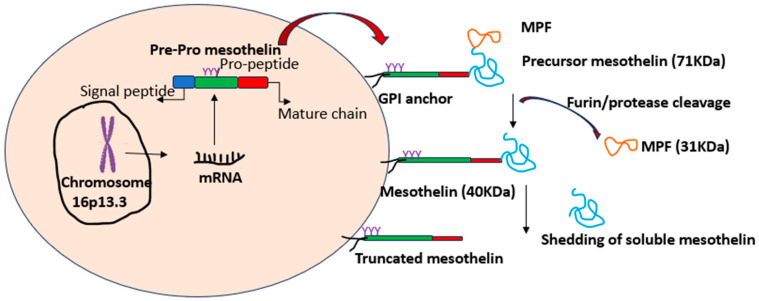
MSLN structure and processing: The MSLN gene is located on chromosome 16p13.3, which encodes a 622-residue precursor polypeptide known as pre-pro-mesothelin. During processing, the signal peptide is removed and the precursor undergoes furin-mediated cleavage at residue R295, generating two distinct products: (1) A 31 kDa secreted megakaryocyte-potentiating factor (MPF) and (2) A 40 kDa glycosylphosphatidylinositol (GPI)-anchored mesothelin. This figure illustrates the MSLN gene structure, precursor processing stages, cleavage sites and post-translational modifications.

Mesothelin is a glycoprotein abundantly expressed in lung, ovarian, colon, pancreatic and mesothelioma cancer and due to its distinctive expression pattern and key role in enhancing cancer dissemination, aggressiveness and drug insensitivity, MSLN is an intriguing marker for cancer therapy. Despite its immense potential in cancer progression, the complicated regulatory and physiological functions of MSLN remain unexplored. This review provides an in-depth overview of the biological function of MSLN and up-to-date clinical significance [1]. The MSLN gene, with 17 exons, is located on chromosome 16p13.3 and generates the 622-residue precursors’ polypeptide, referred to as pre-pro mesothelin. During the several processing stages, this precursor protein passes through and the signal peptide and is eliminated. The glycosylphosphatidylinositol (GPI) anchors MSLN with furin-mediate cleavage at residue R295. Two different products result from this cleavage: a 31-kilo Dalton-secreted megakaryocyte-potentiating factor (MPF) and a 40-kilo Dalton GPI-anchored mesothelin [2]. While furin is the most significant enzyme involved in this process, additional proteases can also digest mesothelin, albeit at a slower rate. Additionally, residues N388, N488 and N515 on mesothelin include three possible N-glycosylation sites [3], as shown in Figure 1.

A 20-base-pair promoter region, termed as Canscript, includes transcriptional factors MCAT and SP1-like components, which control the expression of MSLN. The expression levels of mesothelin are regulated by the MCAT element’s binding to transcription enhancer factor-1 (TEF-1). Mesothelin transcription is also controlled by epigenetic regulation, including promoter methylation and splicing variants; however, in resilient cells, demethylation may elevate mesothelin expression further. In addition, mesothelin transcription is also modulated by Wnt proteins (Wnt-1) and microRNAs, such as miR-21 and miR-198. For instance, MSLN and miR-198 have an inverse relationship where miR-198 functions as a tumor suppressor by regulating genes involved in cell proliferation, migration and survival. In normal cells, miR-198 levels are maintained to prevent uncontrolled growth. However, in cancers, mesothelin is often overexpressed, leading to the downregulation of miR-198. This suppression occurs through pathways like NF-κB and PI3K/AKT, which are commonly activated in tumors. Thus, restoring miR-198 expression can downregulate mesothelin, inhibiting tumor progression [4]. Thus, to completely understand how Wnt/beta-catenin signaling regulates mesothelin, further extensive investigation is required [4]. The association of MSLN with another glycoprotein, CA-125 (MUC16), facilitates cell adhesion. The 64 residues at the N-terminal of MSLN is the precise location of the CA-125 binding site, which has been determined through stringent study methods such as truncate and alanine substitutions mutation. It is noteworthy that this region’s glycosylation position influences the binding affinity. The interaction between MSLN-CA-125 can protect tumor cells from immune responses and enhance their survival. Both MSLN and CA-125 are being explored as therapeutic targets, with approaches including monoclonal antibodies, antibody-drug conjugates and CAR-T cell therapies to disrupt their interaction and inhibit tumor growth [5]. Furthermore, varying degrees of tumoricidal impact are shown by CAR-T cells engineered to target the glycosylated C-terminal (487–598) or N-terminal (296–390) parts of MSLN, emphasizing the vital function of MSLN glycosylation has in regulating T-cell recognition and tumor suppression [2]. Additionally, in stress, MSLN exhibits total dominance over proliferation and fibrogenic activation by forming a signal complex with CA-125 and Thy1 [2]. Thus, MSLN plays a critical role in cancer progression and its interaction with CA-125 worsens the condition.

## 2. Mesothelin in Normal and Cancer Cells

Epithelial cells of the trachea, tonsils, fallopian tubes and testicular tunica vaginalis, mesothelial cells of the pleura, peritoneum and pericardium are the tissues where mesothelin is primarily expressed [1]. The critical mechanisms like cell adhesion, cellular interactions and tissue structure maintenance are primarily facilitated by MSLN, though its precise physiological role is still unknown [6]. Moreover, MSLN could potentially be associated with immune response and inflammation regulation based on the results that have emerged from a thorough examination of various biomarkers, including soluble MSLN-related levels of proteins, MSLN methylation and inflammatory markers [7].

MSLN plays a vital role in survival, proliferation and apoptosis; its suppression enhances chemosensitivity and its inability to bind to CA-125 reduces drug sensitivity. Additional investigation is required to understand its pathobiological mechanisms and therapeutic and diagnostic potential due to its crucial role in cancer progression and dissemination, regulation of the EMT and also controlling the cancer stem cells. Poor patient outcomes and relapse-free survival have been associated with MSLN expression in gastric, pancreatic and ovarian cancer, cholangiocarcinoma, breast cancer, endometrial cancer and lung cancer [8,9]. MSLN networks with a cancer antigen CA125/MUC16 trigger a chain of signaling events, which in turn suppresses a Dickkopf-1 (DKK1), a secreted protein that inhibits the Wnt signaling pathway and stimulates cell migration. By hindering this cascade reaction, the DKK1 levels can be replenished in ovarian cancer [10]. Elevated levels of MSLN expression are identified in pancreatic cancer cells exhibiting chemoresistance, causing enhanced adhesion of cells, proliferation, migration and invasion. MSLN knockout, however, diminished these traits. MSLN overexpression enhances resistance to the chemotherapeutic drug gemcitabine since it promotes the EMT and characteristics found in cancer stem cells. To reverse EMT and chemoresistance in pancreatic cancer cells, targeting MSLN may be a viable strategy [8].

## 3. MSLN in Epigenetics and Signal Pathways

Since MSLN lacks an intracellular binding domain, it is not a classic driver of oncogene, but it is considered an oncogenic accessory protein or a tumor-associated antigen. Its expression is often upregulated in cancers and correlates with poor prognosis. It interacts with receptors, enzymes, or other proteins, promoting tumor growth, invasion, immune evasion and metastasis [11]. The EMT is a crucial stage in the invasion and metastasis of solid tumors where there is an elevated mesenchymal and stemness marker and decreased epithelial marker expression, which can be related to MSLN expression. MSLN’s involvement as cancer hallmarks is highlighted by the reduction in carcinoma development, migration, invasion, metastasis and anchorage-independent growth shown in lung cancer cells following MSLN knockdown [12]. Furthermore, MSLN knockdown decreases the stem cell quality and reverses EMT [9]. MSLN expression has been shown to heighten tumor aggressiveness, albeit this impact is not exclusively attributable to its interaction with MUC16. To explain the features of MSLN-positive tumors both *in vitro* and *in vivo*, numerous investigations have been performed to analyze cellular signal pathways. Although a specific mechanism could not be highlighted suppressing MSLN inhibited cell growth and tumor proliferation in vivo [5]. Malignant pleural mesothelioma is highly aggressive with poor prognosis. Serum level of mesothelin-related peptide analysis of MPM tumors recognized three CpG methylation location in the MSLN promoter, which is located 214 bp upstream of the transcription start point. Notably, the methylation levels at these CpG sites were significantly higher in normal pleural tissue compared to tumor tissue (*p* < 6.0 × 10⁻⁹). This suggests that hypomethylation, or the loss of methylation, in the MSLN promoter region may contribute to the overexpression of mesothelin in MPM tumors. Furthermore, tumors from SMRP-negative patients showed higher MSLN methylation than SMRP-positive patient [13]. These conclusions suggest that MSLN is typically methylated in normal pleura but undergoes demethylation in most MPM tumors, which causes mesothelin overexpression. However, a subset of tumors retains methylation, explaining the SMRP assay’s reduced sensitivity. Also, histoepigenetic analysis classified tumor samples based on intrinsic MSLN expression, revealing discrete subgroups in PDAC and MPM, further supporting the role of epigenetic regulation in mesothelin expression [14]. These insights may help refine biomarker strategies to improve MPM screening, particularly among high-risk populations.

**Figure 2:**
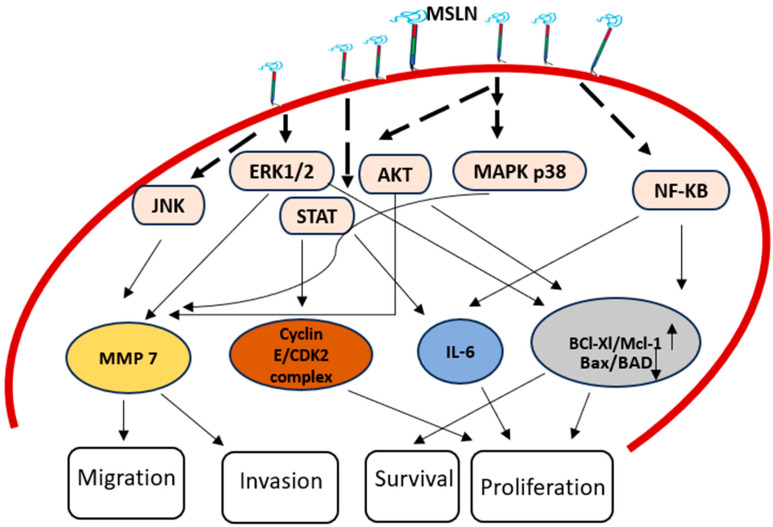
MSLN in cancer progression: mesothelin overexpression contributes to cancer progression by modulating multiple signaling pathways. MSLN enhances cyclin E expression, promoting cell proliferation and cell cycle progression via STAT3 activation. MSLN activates NF-κB, leading to the production of interleukin-6 (IL-6), leading to cell growth and survival. MSLN also activates the AKT pathway, which increases BCl Xl/Mcl1 and decreases Bax/BAD, leading to cancer progression. MSLN promotes the production of MMP-7 through ERK1/2, Akt and JNK signaling pathways, enhancing cell motility and invasion.

MSLN augments the expression of cyclin E, accelerating cell proliferation and the cell cycle through the activation of STAT3. Furthermore, it is demonstrated that MSLN activates the process that leads to the activation of NF-κB, which in response, produces interleukin (IL)-6, which promotes cell growth and survival. Also, MSLN has been demonstrated to influence OCT-2 and NF-κB, resulting in the reduced expression of the tumor suppressor miR-198 [15]. MSLN interacts through the hormone RARG and tyrosine TNK2 to trigger AKT, owing to an *in-silico* strategy that used the cancer genome atlas (TCGA) [16]. This has implications in the Wnt signaling pathways and the development of cancer [17]. Utilizing the ERK1/2, Akt and JNK signaling pathways, MSLN interaction to surface- tethered MSLN promotes the production of MMP-7, thereby enhancing cell motility and invasion. MMPs serve as crucial regulators of cell behavior, influencing motility, development, differentiation and mortality. Alterations in MMP expression have been linked to several cancer types. Elevated MMP-7 levels have been associated with the overexpression of MSLN in ovarian cancer cells, which may be triggered by the increased solubility of MSLN or trans-interactions between MSLN molecules on adjacent cells, as depicted in Figure 2. Additionally, in pancreatic ductal adenocarcinoma, MSLN attaching to CA125/MUC16 also stimulates MMP-7 manifestation via the p38 MAPK pathway [18]. Table 1 list various signal pathways influenced by MSLN.

## 4. MSLN-Targeted Therapies

Targeted therapy identifies and targets cancer cells by targeting proteins, genes, or factors involved in cancer growth, while minimizing damage to normal cells. It can be used alone or with other treatments like chemotherapy, surgery or radiation. Targeted therapies block signals that trigger cancer cell growth destroy proteins, prevent angiogenesis and stimulate the immune response. Target therapies include small or large-molecule drugs, with small molecules entering cells and large molecules targeting surface proteins, for instance, the use of angiogenesis inhibitors, monoclonal antibodies, proteasome inhibitors and signal transduction inhibitors. Some targeted therapies also act as immunotherapies by enhancing immune responses.

(a) The combination treatment unequivocally utilizes CAR-NK cells that are MSLN-specific for ovarian cancer.

Metastatic ovarian cancer exhibited enhanced concurrent expression of MSLN and CA125 which is a noteworthy correlation between the manifestation of MSLN and CA125 as markers and the cancer progression. MSLN- and CD19-CAR NK cells have been employed to evaluate the therapeutic efficacy of CAR-modified NK cells targeting MSLN in ovarian cancer. Notably, MSLN and CD19-CAR NK cells exhibited a negligible impact on SK-HEP-1 MSLN-negative cells, while decisively eliminating OVCAR-3 and SK-OV-3 MSLN-positive ovarian cancer cells in vitro studies. Furthermore, when co-cultured with OVCAR-3 and SK-OV-3, MSLN-CAR NK cells unequivocally generated substantially more cytokines than parental NK-92 cells and CD19-CAR NK cells. Additionally, by eradicating ovarian cancer, MSLN-CAR NK cells led to a substantial improvement in the survival ratio of mice with intraperitoneal malignancies. These remarkable findings demonstrated that MSLN is a very promising target for ovarian cancer treatment using CAR NK cell therapy [19].

A research study investigated the effectiveness of combining MSLN-TTC (MSLN-targeted 227Th conjugate, BAY 2287411) with various DDR inhibitors targeting distinct enzymes in the DDR pathway. In vitro experiments assessed the cytotoxic effects of combining MSLN-TTC with inhibitors of ATM, ATR, DNA-PK and PARP 1/2. The results showed synergistic effects with all inhibitors, with the most pronounced enhancements observed with ATRi and PARPi. Furthermore, in vivo studies using OVCAR-3 and OVCAR-8 xenograft models demonstrated significant synergistic anti-tumor activity when combining MSLN-TTC with ATRi, even at doses that were ineffective as monotherapies. These findings suggest potential therapeutic benefits from combining MSLN-TTC with DDR inhibitors, particularly ATRi [20].

(b) Introducing CAR-T cells targeting MSLN in co-treatment with proton radiation therapy: The co-expression of MSLN in pancreatic ductal adenocarcinoma (PDAC) can promote tumor development, metastasis and worse patient outcomes. Intraductal papillary mucinous neoplasm (IPMN) immunohistochemical investigation demonstrated that MSLN expression existed in cancer and adenoma cells, but not in normal pancreatic tissues [21]. Amit et al. (2024) demonstrated RT and MSLN-targeting CAR-T cell therapy, combined in a PDAC model, have been demonstrated to elevate MSLN expression in cancer cells, enhance CAR-T cell infiltration into malignancies, alter immunosuppressive M1 macrophages to anti-cancer M1 macrophages and minimize MDSCs. In the flank PDAC model, the combined therapy established more malignancy inhibition and improved survival, while in an orthotopic PDAC model, it substantially decreased tumor growth and development and in a dual-flank tumor model, it had abscopal effects. Furthermore, it led to enhanced extratumoral CAR-T cell proliferation and elevated serum interferon-γ levels. It has been suggested that RT, in conjunction with MSLN-targeting CAR-T treatment, represents a viable strategy for enhancing tumor microenvironment modulation, CAR-T cell trafficking and anti-cancer effects in patients with unresectable PDAC [22].

(c) Developments in colon cancer imaging and CAR-T cell therapy: An investigation evaluated the implementation of a 124I-labeled MSLN antibody for non-invasive MSLN (MSLN) expression detection in colon cancer. With accurate and high affinity binding to the MSLN protein, the radiotracer demonstrated excellent radiochemical purity and stability. Comparing MSLN-overexpressing LS174T cells and malignancies to control cells and tumors revealed significantly higher absorption in the first group. Along with having significant tumor-to-muscle ratios, low uptake by organ and high uptake by tumor, the radiotracer also showed positive biodistribution and dosimetry profiles. These results demonstrate the potential of 124I-anti-MSLN as an effective tool for the diagnosis of tumors overexpressing MSLN support its continued advancement for use in cancer imaging and treatment [23].

Nonetheless, in the immunosuppressive tumor milieu, fused antigen receptor (CAR) T-cell therapy has limited effectiveness against dense tumors as adenosine is a major factor in the suppression of T-cell responses by activating adenosine 2A receptor (A2aR). Thus, short hairpin RNA (shRNA) is used to interact with A2aR in CAR-T cells that are anti-MSLN (MSLN). MSLN-positive ovarian and colon cancer cells were used in vitro studies demonstrating markedly improved CAR-T cell activity with A2aR disruption, including increased cytokine generation and cytotoxicity. Related to non-transduced or wild-type CAR-T cells, A2aR-disrupted CAR-T cells obtained a higher tumor burden reduction in vivo investigation employing SKOV3 xenograft mice models. These results imply that a viable strategy to increase CAR-T cells is shRNA-mediated A2aR disruption [24].

By activating inhibitory receptors, like PD-1, through ligands like PD-L1, tumors thwart CAR-T cells and assault and cause T cell fatigue. To address this, MSLN-expressing ovarian (SKOV3) and colon (HCT116) cancer cells were used to study the impact of PD-1-mediated fatigue on MSLN-targeted CAR-T cells. PD-1 expression was significantly reduced by incorporating PD-1-targeting shRNA into CAR-T cells. This reduction greatly improved the performance of CAR-T cells, including the capability to produce cytokines and being cytotoxic to PD-L1-positive cancer cells performed in *in vitro* studies. The findings demonstrate that MSLN-targeted CAR-T cells with PD-1 silencing have enhanced anti-tumor activity, indicating that silencing genes to target particular immune checkpoints may enhance CAR-T cell therapy for various types of cancer [25]. The study analyzed MSLN expression in 270 primary and 44 metastatic CRC cases. It was found that MSLN manifest was related to the female gender and inversely correlated with solid/sheet-like tumor proliferation. Diffuse luminal/membranous MSLN expression was linked to shorter overall survival and identified as a potential risk factor through multivariable analysis. Studies have demonstrated that MSLN triggers colon cancer progression; thus, MSLN immunohistochemistry is a powerful prognostic tool for colorectal carcinoma and anti-MSLN therapy could be effective in treating MSLN-positive CRC, even at advanced metastatic stage [25,26].

MSLN manifest in CRCs is linked to poor prognosis. MSLN expression in 314 stage II CRC patients using tissue microarrays and immunohistochemistry. A deep learning algorithm ensured objective analysis showed a high correlation between hands-on and automated-learning MSLN staining ratios (r = 0.71), significant correlations between different tumor regions (Ro vs. Ce, r = 0.63; Ro vs. Fr-sm, r = 0.54; Ro vs. Fr-ss, r = 0.61) and disease-specific survival rates differed significantly between MSLN-positive cohort and MSLN-negative cohort in various regions (*p* = 0.024, 0.0087, 0.051 and 0.046). Thus, MSLN expression is relatively uniform within stage II CRC tumors and high expression indicates a poorer prognosis, regardless of tumor area [27]. Colorectal cancer in stage IV has a high expression of MSLN, which is correlated with chemo-resistance, aggressiveness and deprived prognosis. However, in pancreatic adenocarcinoma, although MSLN is commonly expressed, it does not correlate with cancer aggressiveness, indicating that MSLN’s expression and its impact on prognosis vary by cancer type [18].

## 5. Clinical Trials Involving MSLN-Targeted Treatments

Since cell surface MSLN has shown promise as a target for immunotherapy, several therapeutic drugs targeting this protein are presently being developed and assessed in preclinical and clinical trials [28]. A modified form of *Pseudomonas* exotoxin A and a mutable fragment (Fv) of SS1 combines the recombinant agent immunotoxin SS1POne. SS1P has proven to be safe and effective against tumors in phase I scientific trials performed at the National Cancer Institute (NCI) in the United States, with certain limitations like infusion-related reactions such as fever, chills and hypotension, capillary leak syndrome, i.e., fluid leakage causing edema, low blood pressure and potential organ damage, as well as SS1P may stimulate the immune system to produce anti-drug antibodies, reducing its therapeutic effectiveness over time [29]. Leveraging on these findings, SS1P, in conjunction with chemotherapy, is currently being studied in a new clinical experiment. A noteworthy therapeutic drug is MORAb-009, also known as amatuximab. It is a chimeric antibody that blends human IgGγ1 and k constant sections with murine SS1 Fv. In a Phase I trial, 24 patients (thirteen mesothelioma, seven pancreatic cancers and four ovarian cancer) received MORAb-009, with a median of four infusions (range 1–24). Dose-limiting toxicities (DLTs) occurred at 400 mg/m^2^, leading to the maximum tolerated dose (MTD) of 200 mg/m^2^. Adverse events included grade 1–2 hypersensitivity (seven cases) and one grade 4 thromboembolic event. Eleven patients achieved stable disease and serum MORAb-009 concentrations increased dose-dependently [30]. Novel antibodies are currently under thorough investigation for their potential therapeutic applications, complementing existing compounds. One such antibody, m912, has been meticulously isolated from a human Fab library. The hmab shows great promise by inducing antibody-dependent cellular cytotoxicity (ADCC) and binding specifically to cell surface MSLN. Utilizing phage display expertise, a single-chain mutable fragment (scFv), known as HN1, has been engineered as the basis for developing a high-affinity human mAb, which demonstrates robust ADCC and effectively targets and eliminates cancer cells expressing MSLN with remarkable affinity (KD = 3 nM) [31].

Moreover, a recombinant immunotoxin was constructed with potent cytotoxic activity in the contradiction of cancer cells through the strategic fusion of the HN1 scFv with a truncated PE. Thus, HN1 and the immunotoxin it produces, hold immense potential for revolutionizing the treatment of MSLN-expressing cancer. In 126 patients with particular tumors, a phase I/IIa trial (NCT02341625) investigated the efficacy and tolerance of BMS-986148, an MSLN-directed ADC, either in addition to nivolumab or on its own. Elevations in liver enzymes were remarkably prevalent as treatment-related adverse events (TRAEs). Among patients receiving BMS-986148, 49% experienced grade 3/4 TRAEs when administered every three weeks, 25% when received weekly and 33% in combination with nivolumab. Additionally, 13% of patients have obsolete treatment due to TRAEs. It was determined that the maximum tolerated dosage (MTD) every three weeks was 1.2 mg/kg and the safety profile remained consistent when nivolumab was incorporated. The study observed preliminary clinical action, with no discernible correlation between response and MSLN expression. Based on the study’s findings, BMS-986148 + nivolumab exhibits a controllable safety profile and presents the potential for further exploration as a multimodal therapy for advanced solid tumors [32].

CAR-T cells directing MSLN have demonstrated promising outcomes in preclinical studies, demonstrating a significant tumor reduction. Ongoing clinical trials are focusing on factors like their safety and efficacy in solid tumors. The initial Phase I data indicates limited clinical efficacy but tolerability with few off-target effects. T cell receptor fusion constructions (TRuCs), a novel T cell engineering platform, have demonstrated strong anti-tumor efficacy, enhanced tumor accumulation and long-term persistence, indicating significant progress in MSLN-targeted immunotherapy [33]. Increased anti-tumor effects and decreased antigen escape have been observed with tandem CAR-T cells that target manifold antigens, including MSLN and folate receptor 1 (FOLR1). Silencing PD-1 and Tim3 genes in CAR-T cells has also improved efficacy and cytotoxicity. Additionally, knocking down adenosine 2a receptor (A2aR) has increased anti-tumor ability. Engineering CAR-T cells with chemotactic receptors like CCR2b and CCR4 has enhanced migration and cytotoxicity. These advancements offer promising strategies to overcome tumor microenvironment limitations and improve CAR-T cell therapy outcomes. To create more potent and long-lasting CAR-T cell treatments to combat various malignancies, researchers focus on targeting several antigens and modifying gene expression [34]. FOLR1 and MSLN are highly expressed in cancer tissues, with only 89% of samples positive for both. While targeting these antigens individually achieves a 48–76% tumor elimination rate, simultaneous targeting increases tumor cell killing to 88% in ovarian cancer. Third-generation TanCAR targets both FOLR1 and MSLN, coupled with IL-12 secretion, to reduce antigen escape and enhance CAR-T cell infiltration. Additionally, with 80% of epithelial ovarian cancers expressing mucin 16 (MUC16) and the inhibitory PD-1/PD-L1 signaling pathway impairing T cell immunity, a TanCAR targeting PD-L1 and MUC16 has been proposed. Although initial studies showed no significant differences between TanCAR-T cells and other CAR-T therapies, further optimization may enhance efficacy [35]. Improved implementation of CAR-T cell treatment for NSCLC by a combination of MSLN-specific CAR (Msln-CAR) alongside the CCR2b or CCR4 chemokine receptors. In addition to enhancing cytotoxicity and cytokine production versus MSLN-positive tumor cells, this combination improved CAR-T cell motility in vitro. In an NSCLC xenograft model, Msln-CCR2b-CAR-T cells showed superior anti-tumor activity, greater migration and permeation into tumor tissue, without causing significant organ damage. As a potential application, the study focuses on the efficacy of combining chemokine receptors with CAR-T cell therapy to enhance therapy outcomes for NSCL cancer [36]. Bispecific T cell fusion proteins termed engagers (BiTEs) improve the cytotoxicity of T cells against cancer. In solid tumors such as TNBC, pancreatic ductal adenocarcinoma and lung cancer, MSLN-targeting BiTEs are presently being explored. Modification, including adding an Fc-domain or serum albumin-binding capabilities, to prolong the half-life of BiTEs and minimize the frequency of dosage to overcome their short half-life. Promising outcomes from preclinical research include enhanced effectiveness and uptake specific to tumors. A trispecific construct, HPN536, is a possible novel therapy strategy for solid tumors and has started a phase I clinical study (NCT03872206) [37].

Furthermore, by utilizing the connection between MUC16 and MSLN, a unique human immune adhesion called HN125, which specifically targets tumor-associated MUC16, was developed [38]. This improvement showed promising results for ovarian cancer and other MUC16-expressing tumors, by efficiently inhibiting the MSLN-MUC16 interaction and triggering ADCC (antibody-dependent cell-mediated cytotoxicity) against ovarian tumor cells. Meanwhile, MSLN-targeted monoclonal antibodies are not efficient in halting the growth of cancer cells [39]. This may be because many of these antibodies target MSLN’s N-terminal Region I instead of the crucial signal regions. Region I is highly immunogenic, making it challenging to develop antibodies that target other domains. Antibodies directed against Region I are unable to prevent the proliferation of cancer cells producing MSLN [39].

Tumor cell vaccine GVAX stimulates anti-tumor effect by expressing numerous antigens, including MSLN. Based on this concept, CRS-207, a Listeria-based vaccine was developed to target MSLN. CRS-207, which presents MSLN to immune cells via MHC, elicits an immune response. Phase II trial results showed improved survival in PDAC cases; phase IIb also had similar results in survival rates when combined with chemotherapy. Phase I clinical trial results were promising with well-tolerated, reduced tumor size and improved survival in mesothelioma cases, including one comprehensive response [40]. The study is an early-stage, single-arm, open-label trial investigating mesothelin-specific fused antigen receptor T cells (LD013) in cases with intractable or relapsed mesothelin-positive ovarian cancer. It contains two phases: a dose escalation phase to determine the appropriate dose and an extension phase to further assess the safety and efficacy of LD013 in this patient population. To completely comprehend MSLN’s operations and maximize its targeting for the better. MSLN-targeted immunotherapies hold immense potential for revolutionizing cancer therapy, contributing new hope for cases with partial options [41]. Malignant pleural mesothelioma (MPM) is caused by genetic abnormalities and gene mutations in BAP1, CDKN2A, epigenetic regulators (KDM4A, LSD1) and signaling proteins (GRP78, STAT3). Clinical studies with high efficacy, including mesothelin-targeting CAR-T cell therapy, PARP inhibitors (Rucaparib), CDK4/6 inhibitors (abemaciclib), histone methyltransferase inhibitors (Tazemetostat) and TEAD inhibitors (VT3989, IK-930) are being focused [42]. Although fused antigen receptor T cell treatment has demonstrated efficacy in blood malignancies, dense tumors due to the immunosuppressive microenvironment are less effective. Thus, CAR-IL12R54 T cells have been advanced to improve their efficacy as they secrete an IL-12 fusion protein and target mesothelin (MSLN). Likened to conventional CAR-T cells, these modified T cells showed efficient tumor lysis in invitro studies and suppressed tumor development in vivo with fewer adverse effects. The durability of anti-tumor responses was obtained when PD-1 inhibition was combined with CAR-IL12R54 T cells. Investigations demonstrated increased production of IFN-<0x7B>, decreased activity of regulatory T cells and improved infiltration and persistence of T cells, providing a potentially effective treatment for solid tumors [43]. Table 2 provides the list of clinical trial for MSLN based therapeutics.

## 6. Conclusions

Finally, given that MSLN has been linked to both the development and metastasis of cancer, it is a viable target for cancer treatment. Targeting it for therapeutic and diagnostic purposes is desired due to its complex regulation and interactions with other molecules. The treatment of cancer has shown a notable improvement using MSLN-based targeted medicines. Chimeric antibodies like MORAb-009, recombinant immunotoxins like SS1P and new medicines like HN1 and m912 have all demonstrated encouraging outcomes in recent times for MSLN-targeted cancer therapy. Preclinical and clinical studies indicate promise for CAR-T cell treatments and bispecific T cell engagers (BiTEs). Moreover, mesothelioma and pancreatic cancer patients are exhibiting higher survival rates because vaccinations like CRS-207, which targets MSLN.

## Figures and Tables

**Table 1:** The role of MSLN in signal pathways.

Signal Pathways	Mechanism
NF-κB Pathway	Augmented expression of MSLN triggers NF-κB, causing augmented expression of IL-6, which in turn promotes cancer survival and proliferation
IL-6/sIL-6R Trans-Signaling Pathway	MSLN helps formation of an extracellular complex between IL-6 and sIL-6R, which triggers trans-signal pathway called the IL-6/sIL-6R pathway which in turn increases the survival and growth of cancer.
Stat3 Pathway	MSLN activates Stat3, increasing cyclin E expression and accelerating cell cycle advancement from G1 to S phase
ERK1/2 Pathway	Pro-apoptotic factor Bim is repressed by MSLN’s persistent ERK1/2 activation, which cause anoikis insensitivity.
MAPK Pathways (p38 MAPK, ERK and JNK)	MMP-7 and MMP-9 expression are triggered by MSLN, causing increased cancer invasion and migration.
p53 Pathway	Inhibiting apoptosis and promoting cell proliferation, MSLN modifies the p53-dependent regulation of apoptosis-related proteins.
β-Catenin Pathway	Reducing MSLN expression has an impact on invasion and EMT by lowering β-catenin expression.

**Table 2:** List the clinical trials for MSLN-based therapy.

Trial Number	Status/Year	MSLN-Based Therapy	Clinical Trials	Ref
NCT05779917	Active/2024	mesothelin-targeted chimeric antigen receptor (CAR) T cells. Engineered to express a fusion protein of IL21 and scFv against PD1.	A Phase I study to evaluate the safety, tolerance and preliminary efficacy of second-generation mesothelin-targeted CAR-T cells, engineered to secrete a fusion protein of IL21 and scFv against PD1, for the immunotherapy of mesothelin-expressing cancers.	[44]
NCT01445392	Terminated/2011	SS1P (recombinant immunotoxin)	Phase I trials showed safety and anti-tumor efficacy, leading to a new trial combining SS1P with chemotherapy.	[45]
NCT00325494	Completed/2009	MORAb-009 (amatuximab, chimeric antibody)	Phase I trial demonstrated stable disease in 11 patients, with ongoing Phase II trials	[46]
NCT01417000	Completed/2014	GVAX	Presents MSLN to immune cells via MHC elicits an immune response	[47]
NCT01439152	Completed/2019	BMS-986148	Phase 1 clinical trials. MSLN-directed antibody-drug conjugate (ADC), alone or with nivolumab, in 126 patients with selected tumors	[48]
NCT05397093	Active/2022	FOLR1	Targeting multiple antigens and modulating gene expression	[49]
NCT02810418	Completed/2016	LMB-100	LMB-100 is given in combination with tofacitinib to patients with pancreatic adenocarcinoma, extrahepatic cholangiocarcinoma and other MSLN-positive solid tumors	[50]
NCT02639091	Completed/2020	Anetumab Ravtansine in Combination With Pemetrexed and Cisplatin	In Phase 1b, BAY 94-9343 in combination with pemetrexed and cisplatin was administered to the patient with metastatic epithelial mesothelioma or nonsquamous non-small-cell lung cancer (NSCLC). To study was to assess the assessing the safety, tolerability and efficacy of the drug regimen. Antibody-drug conjugate (ADC) is directed against the cancer antigen MSLN on tumor cells.	[48]
NCT02159716	Completed/2014	CART-meso in Mesothelin Expressing Cancers	Phase I Study of Chimeric Antigen Receptor Modified T Cells in Patients With Mesothelin Expressing Cancers	[51]
NCT03872206	Completed/2023	HPN536	Phase 1/2a study of HPN536 as monotherapy to assess the safety, tolerability and PK in patients with advanced cancers associated with mesothelin expression	[37]
NCT05372692	Completed/2023	Mesothelin-specific Chimeric antigen Receptor T Cells (LD013)	Chimeric antigen receptor T cells (LD013) in patients with refractory or relapsed mesothelin-positive ovarian cancer	[41]
NCT02884726	Completed/2020	BMS 986148	Phase 1 Study of Mesothelin-ADC Study is to assess the safety and tolerability of Mesothelin-ADC in subjects with advanced and/or metastatic solid tumors	[32]
NCT01675765	Completed/2018	CRS-207 attenuated form of Listeria monocytogenes	Clinical trial assesses the safety and immune response of CRS-207, a modified Listeria monocytogenes cancer vaccine targeting mesothelin, given with or without cyclophosphamide, followed by pemetrexed and cisplatin.	[52]
NCT02341625	Terminated/2020	BMS-986148 a drug	Combination treatment of BMS-986148 (a drug) with or without nivolumab an immune checkpoint inhibitor a safe and tolerable. Some patients experienced long-lasting positive responses to the treatment.	[32]

## Abbreviations

ATM	Ataxia telangiectasia mutated
ATR	Ataxia telangiectasia and Rad-3-related protein
DNA-PK	DNA-dependent protein kinase
ADC	Antibody-drug conjugate
MDSCs	Myeloid-derived suppressor cells
MCAT	Muscle-CAT binding site
MMP-7	Matrix metalloproteinase-7
NF-κB	Nuclear factor kappa B
NSCLC	Non-small-cell lung cancer
PE	Pseudomonas exotoxin A
RARG	Retinoic acid receptor gamma
RT	Proton radiation therapy
SP1	Specificity Protein 1
STAT 3	Signal transducer and activator of transcription 3
TNBC	Triple-negative breast cancer
TNK2	Kinase non-receptor 2
